# Gestion de l’anémie des patients hémodialysés chroniques: cas du Service de Néphrologie et d’hémodialyse du CHU du Point G au Mali

**DOI:** 10.11604/pamj.2017.26.167.10861

**Published:** 2017-03-23

**Authors:** Alhadji Ahmadou Tounkara, Abdoul Mahama Sériba Coulibaly, Nouhoun Coulibaly, Békaye Traoré, Mahamane Kalil Maïga

**Affiliations:** 1 Service de Néphrologie et d’Hémodialyse du CHU du Point G, Bamako, Mali; 2 Centre National d’Appui à la Lutte Contre la Maladie, Bamako, Mali

**Keywords:** Anémie, hémodialyse, EPO, transfusion sanguine, pays pauvre, Mali, Anemia, haemodialysis, EPO, blood transfusion, Poor country, Mali

## Abstract

**Introduction:**

L'anémie est une complication fréquente de l'IRC couramment retrouvée chez les patients hémodialysés chroniques. Chez ces derniers, la prise en charge est principalement basée sur l'administration d'érythropoïétine et la supplémentation en fer. Le but de ce travail était d'évaluer la prise en charge de l'anémie des hémodialysés chroniques dans le service de Néphrologie du CHU du Point G.

**Méthodes:**

Il s'agissait d'une étude transversale réalisée dans ledit service du 1^er^ au 31 Août 2016.

**Résultats:**

Au total, 63 patients sur 174 participants avaient été retenus, 34 hommes et 29 femmes avec un sex-ratio à 1,17 en faveur des hommes. L'âge moyen des patients était de 48,79 ans ± 11,59, la durée moyenne en hémodialyse était de 3,77ans ± 2,6. La fréquence hospitalière de l'anémie chez nos dialysés était de 84,12%. La transfusion sanguine était retrouvée chez 92,1%, avec une moyenne annuelle de 5,81poches ± 5,91. La supplémentation martiale était notée dans 87,3% des cas. Les moyennes respectives de ferritine et de CST étaient de 1245 ng/ml ± 629,52 et 46,16%±19,12. L'administration occasionnelle d'EPO à des doses n'excédant pas les 4000UI était rapportée par 79,4% des patients. La principale difficulté pour l'utilisation de l' EPO était le coût (74,6%). L'infection au VHC touchait 60,1% des patients ayant réalisé le dit bilan.

**Conclusion:**

La gestion de l'anémie des dialysés chroniques doit être intégrée dans un cadre politique nationale de la santé.

## Abstract (en)

**Introduction:**

Anemia is a frequent complication of Chronic Kidney Disease CKD commonly found in chronic haemodialysis patients. Patients management is mainly based on erythropoietin administration and iron supplementation. This study aimed to evaluate the management of chronic hemodialysis patients with anemia in the Department of Nephrology and Hemodialysis at the University Hospital Point G in Mali.

**Methods:**

We conducted a cross-sectional study from 1 to 31 August 2016.

**Results:**

63 patients out of a total of 174 participants were selected, 34 men and 29 women with a sex-ratio of 1.17 in favor of men. The average age of patients was 48,79 years ±11.59, the average duration of hemodialysis treatment time was 3,77years±2.6. Hospitalization rate for anemia in our dialysis patients was 84,12%. 92.1% of patients required blood transfusion, with an annual average of 5,81 blood bags ±5.91. 87.3% of cases required iron supplementation. Mean ferritin concentration and TSC were 1245 ng/ml±629,52 and 46,16%±19.12 respectively. Occasional administration of EPO doses not exceeding the 4000IU was reported by 79.4% of patients. The main difficulty in using EPO was its cost (74.6%). HCV infection was found in 60.1% of patients who were assessed.

**Conclusion:**

The management of chronic dialysis patients with anemia should be integrated into the framework of the national health policy.

## Introduction

L'OMS définit l'anémie comme un taux d'hémoglobine(Hb) inférieur à 12g/dl chez la femme ou inférieur à 13g/dl chez l'homme [1]. C'est un problème de santé publique affectant environ 7,6 % de la population générale et atteindrait 60 à 80% des patients souffrant d'insuffisance renale chronique(IRC) [2, 3]. En dialyse itérative, comme au cours de l'IRC, l'anémie est principalement due à une diminution de la production rénale d'érythropoïétine(EPO), à laquelle peuvent s'ajouter la diminution de la durée de vie des hématies, les pertes sanguines, les carences en oligoéléments ou l'inflammation chronique [4]. Sa prise en charge(PEC) a connu depuis la fin des années 80 une nette amélioration avec l'introduction des agents stimulants l'érythropoïèse(ASE), qui ont totalement révolutionné la gestion de cette anémie particulière, diminuant ainsi la fréquence des transfusions sanguines et leurs risques, tout en contribuant à l'amélioration de la qualité de vie des patients [5]. Actuellement, cette PEC est basée sur l'utilisation simultanée d'ASE et la supplémentation martiale, voire en acide folique ou vitamine B12 avec rarement un recours à la transfusion sanguine. Toutes fois, la gestion des risques liés aux doses des ASE, leur coût et les taux d'hémoglobine cibles en fonction des patients restent un problème d'actualité [5, 6]. Notre étude avait pour but d'évaluer de la gestion de l'anémie des hémodialysés chroniques dans notre service.

## Méthodes

Il s'agissait d'une étude transversale menée dans le Service de Néphrologie et d'hémodialyse du CHU du Point G du 1^er^ au 31 Août 2016 concernant tous les patients hémodialysés chroniques dudit service. Les Critères d'inclusion étaient : être hémodialysé chronique du service (plus de 6 mois) et réaliser un bilan dont au minimum une numération formule sanguine (NFS). Avaient été exclus de l'etude, les cas d'hémodialyse pour insuffisance renale aigue (IRA) et les patients n'ayant pas réalisé la NFS. Un questionnaire anonyme avait été rempli individuellement par les patients où les données suivantes ont été recueillies : l'âge, le sexe, la durée en dialyse, la connaissance et l'utilisation de L'EPO et/ou du fer, la notion de transfusions sanguines ainsi que sa fréquence, la moyenne annuelle de poches de sang utilisées. Le bilan demandé était : la NFS, les sérologies de l'hépatite B, C (VHB, VHC) et du virus de l'immunodéficience humaine(VIH), la ferritine et le coefficient de saturation de la transferrine (CST). Nous avons retenu pour nos patients les seuils suivants: comme anémie, tout taux d'Hb <11g/dl, normal si le taux d'Hb est compris entre11-13g/dl et anormalement élevé si le taux d'Hb >13g/dl ; pour le CST : valeur normale si entre 20-40% ; élevée si le CST >40% et basse si le CST < 20% ; pour la ferritine : normale si valeur entre 100-500ng/ml, élevée si la valeur >500ng/ml, et basse si la valeur est <100ng/ml. Les données ont été saisies et analysées avec le logiciel SPSS version 18.

## Résultats

**Figure 1:**
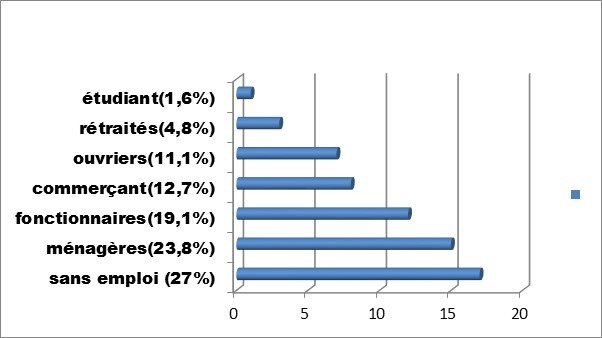
Répartition des patients selon la profession

**Figure 2:**
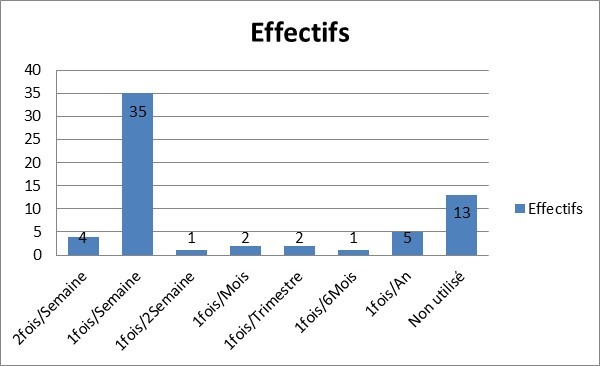
Réapparition des patients selon le mode d’utilisation de l’EPO

Au total, sur les 300 patients que comptait le centre pendant la période d'étude (1er au 31Aouut 2016), 174 patients avaient acceptés de participer à l'étude, parmi lesquels seuls 63 ont pu réaliser le bilan minimum, 34 hommes et 29 femmes, le sex ratio était à 1,17. L'âge moyen des patients était de 48,79 ans ±11,59 ans avec des extrêmes allant de 18 à 70 ans, la durée moyenne en dialyse était de 3,77ans ±2,6ans(extrêmes; 10 mois et 11 ans). La répartition des patients selon la profession exercée est donnée par la Figure 1. Le taux moyen d'Hb des patients était de 8,57g/dl± 2,39 (extrêmes 3,53 et 14,5). Les moyennes respectives de ferritine et du CST étaient de 1245ng/l±629,52 (extrêmes 35,45 à 2000) et 46, 16%±19,12 (extrêmes 16,30 à 91,30). Les patients qui Connaissaient l'EPO représentaient 85,7% contre 14,3% des patients qui l'ignoraient. L'administration occasionnelle d'EPO était rapportée par 79, 4% des patients contre 20,6% des patients qui ne l'avaient jamais utilisé. La Figure 2 illustre les différentes modalités d'utilisation d'EPO par les patients. Dans 74,6% cas les patients trouvaient l'EPO couteux, 19% des patients ignoraient totalement l'EPO et dans 6,3% cas les patients posaient le problème de la disponibilité d'EPO. L'utilisation du fer : absente (12,7%), formes orale et injectable à la fois (7,9%), les formes injectables (66,7%) et les formes orales (12,7%). Le recours à la transfusion était retrouvé chez 92,1% des patients contre 7,9% des patients affirmant ne pas avoir y recours. Parmi les 58 patients transfusés, 43 patients rapportaient une moyenne annuelle de 5,81± 2,91 poches de sang utilisées (extrêmes 2 et 13). La sérologie VIH : négative (28, 6%), positive (1, 6%), non réalisée (69, 8%). La sérologie VHC : négative (17,5%), positive (27%) et non réalisée (55, 6%) La sérologie VHB : négative (26%), positive (1,6%) et non réalisée (57,1%).

## Discussion

Au total, 63 patients sur 174 participants avaient été retenus dans l'etude. L'écrasante majorité de nos dialysés était pauvre, et nombreux étaient ceux qui n'étaient pas parvenus à effectuer le bilan complet demandé dont le coût global s'élevait à l'hôpital national du Point G à 50.000FCFA (≈83 $US). Toutes fois, tous les patients ayant répondu au questionnaire et réalisé la NFS (coût : 1000FCFA ≈1,66 $US) avaient été retenus. L'âge moyen des patients était de 48,79ans±11,59ans pour une durée moyenne en dialyse de 3,77 ans±2,6ans. Les hommes représentaient 54% des patients. Plus de la moitié des patients (50,8%) étaient sans aucune activité régénératrice de revenus (sans emploi et Ménagères). Le taux moyen d'Hb des patients était de 8,57 g/dl± 2,39, seuls environ 15% des patients n'étaient pas concernés par l'anémie. Si la littérature rapportait une prévalence de l'anémie (Hb<11g/dl) chez les dialysés à 75%, malgré une utilisation des ASE [7], dans notre étude cette prévalence était de 84,12%. L'anémie du sujet en IRC est classée comme une maladie endocrine [8], dont les grandes lignes physiopathologiques en hémodialyse se résument surtout à la diminution de la production endogène d'EPO, les pertes sanguines (prélèvements itératifs, gastro-intestinaux, gynécologiques circuits d'hémodialyse), l'inflammation chronique et les etats carentiels (Acide folique, fer vitamine B12). Elle apparait tôt au cours de l'IRC et s'aggrave progressivement avec le déclin de la fonction renale. Sa prise reste basée sur l'utilisation adéquate des ASE, la supplémentation en fer et ou en oligoéléments voire rarement la transfusion sanguine. Cette dernière, est généralement réservée en hémodialyse à certaines situations difficiles (chirurgie, résistance aux ASE), mais restait le premier moyen de PEC de l'anémie des dialysés dans notre étude [9].

En effet, le recours à la transfusion sanguine était quasi-systématique (92,1%) dans notre série avec une moyenne annuelle de poches utilisées par patient à 5,81 poches±2,91. Moyenne ne concernant que 43 patients (sur les 58 transfusés) ayant répondu à la question relative au nombre moyen annuel de poches de sang utilisées, car le centre ne disposait pas de registre à cet effet. La littérature rapporte que l'introduction des ASE dans la PEC de l'anémie des dialysés a diminué de plus de la moitié, les cas de transfusions sanguines [10]. Duclos J et al [11] rapportaient dans leur étude, une réduction des taux de transfusions sanguines en dialyse de 60% à 22% sous ASE. L'usage occasionnel et discontinu d'ASE est retrouvé chez 79,4% contre 20,1% des patients qui ne l'ont jamais utilisé (dont14, 3% par ignorance). Les grandes difficultés rapportées dans l'usage des ASE par les patients étaient surtout leur cherté (74,6%), suivie de l'absence d'information sur les ASE (19%). L'utilisation du fer dans ses différentes présentations, constituait le deuxième mode de PEC de l'anémie selon nos patients. En effet, 66,7% des patients rapportaient utiliser périodiquement du fer injectable et 12,7% des patients utilisaient du fer Per os. L'usage adéquat des ASE reste très peu accessible aux patients des pays en voie de développement comme le Mali où seules les présentations de 2000 et 50000UI d'époetine alpha sont présentes sur le marché à des prix hors de la portée de nos dialysés (5000UI coûte 25000FCFA ≈ 42$US). Aucun patient n'a bénéficié d'une PEC adéquate par ASE (dose d'attaque suivie d'entretien). Gilberson G et al [12], certes dans des proportions moindres, notaient une augmentation des cas de transfusions sanguines aux USA lors de pertes de systèmes d'assurances de leur patients.

Les coûts exorbitants des ASE limitent leur utilisation en dehors de tout système d'assurance. Maoudjoud O et al [13] avaient estimé dans leur etude le coût global par patient et par an des besoins en époetine à 4356,69, une énormité qui exclurait d'emblée nos patients d'un usage adéquat de ses molécules. La logique conséquence, est la supplémentation en fer et la transfusion sanguine, avec une hyperferritininémie > 500ng/ml chez plus de 80% des patients avec une forte prévalence (27%) de l'infection par le virus de l'hépatite C de l'ensemble des patients, soit 60,71% des sérologies testées (17 patients sur les 28 ayant fait le test). Seuls 2 patients VHC positifs ne rapportaient aucune notion de transfusion. L'infection par le VHC affecte environ 2% des populations mondiales [14], mais sa prévalence est plus élevée en HD. C'est un virus facilement transmissible par voie parentérale et la transfusion y joue un rôle majeur en dialyse. Plusieurs études, s'étaient intéressées à sa prévalence en hémodialyse, et cette dernière diffère selon les pays et les centres d'hémodialyse. Senosy SA et al [15] rapportaient une prévalence de 60,9% dans un centre de dialyse en Egypte, tans disque Hasanjani R et al [16], en Iran, rapportaient dans leur etude une prévalence à 8,7% de patients infectés par le VHC. Cette prévalence de l'infection par le VHC parmi nos populations dialysées constitue une véritable menace de santé publique, car en plus de sa transmission par voie sanguine, il se transmet également par voie sexuelle. Or, une étude récemment menée dans le cadre de l'évaluation de la dysfonction érectile de nos dialysés, révélait que seuls 30% des patients n'avaient aucune activité sexuelle [17]. Cela signifie un grand risque pour leur partenaires en général et pour le personnel de dialyse en particulier.

## Conclusion

La gestion de l'anémie du dialysé chronique dépasse le cadre du patient. Elle nécessite une politique volontariste des autorités sanitaires.

### Etat des connaissances actuelles sur le sujet

- L'anémie est une complication de l'insuffisance rénale chronique, courante chez les hémodialysés;
- Sa prise en charge en hémodialyse est surtout basée sur l'administration d'EPO et du fer;
- Les coûts élevés des agents stimulants l'érythropoïèse limitent leur utilisation dans les pays en voie de développement.

### Contribution de notre étude à la connaissance

- Soulève la question du difficile accès des patients dialysés des pays comme le Mali à l'usage adéquat d'érythropoïétine dans le cadre de la prise en charge de l'anémie;
- Appelle les autorités à prendre en compte la menace à l'infection au VHC qui pèse sur nos patients et le reste de la population en termes de santé publique.
